# A Constitutive Relationship for Gravelly Soil Considering Fine Particle Suffusion

**DOI:** 10.3390/ma10101217

**Published:** 2017-10-23

**Authors:** Yuning Zhang, Yulong Chen

**Affiliations:** 1 School of Civil and Architectural Engineering, Yangtze Normal University, Chongqing 408100, China; zhangyuning@yznu.cn; 2 State Key Laboratory of Hydroscience and Engineering, Tsinghua University, Beijing 100084, China

**Keywords:** suffusion, fine particle losses, gravelly soil, constitutive model

## Abstract

Suffusion erosion may occur in sandy gravel dam foundations that use suspended cutoff walls. This erosion causes a loss of fine particles, degrades the soil strength and deformation moduli, and adversely impacts the cutoff walls of the dam foundation, as well as the overlying dam body. A comprehensive evaluation of these effects requires models that quantitatively describe the effects of fine particle losses on the stress-strain relationships of sandy gravels. In this work, we propose an experimental scheme for studying these types of models, and then perform triaxial and confined compression tests to determine the effects of particle losses on the stress-strain relationships. Considering the Duncan-Chang E-B model, quantitative expressions describing the relationship between the parameters of the model and the particle losses were derived. The results show that particle losses did not alter the qualitative stress-strain characteristics of the soils; however, the soil strength and deformation moduli were degraded. By establishing the relationship between the parameters of the model and the losses, the same model can then be used to describe the relationship between sandy gravels and erosion levels that vary in both time and space.

## 1. Introduction

Suffusion describes the phenomenon whereby finer particles erode through the voids between coarse particles due to seepage flow, which is frequently detected in natural deposits and earthen structures. The chronic process of suffusion is always accompanied by the significant dislodgement of soil grains and changes in the hydraulic conductivity, and may result in a loose soil structure because the loss of fine grains will create voids in the coarse matrix. The stress-strain relationship of the suffusional soil may be significantly different from that for the soil without suffusion, and it is likely that the destructive function of suffusion will decrease the strength of the postsuffusion soil.

A classic case of suffusion-induced dam failure is the Hauser Dam in Montana, USA, which collapsed in 1908, one year after its initial impoundment [1]. Richards and Reddy [2] concluded that approximately half of all the dam failures around the world have been related to soil suffusion. In China, a large number of rockfill dams have been constructed directly above deep and thick alluvial strata, and a number of dam sites exhibit especially thick alluvial strata [3]. For technical and economic reasons, many of these dams employ suspended cutoff walls even though the seepage gradient of soils at the bottom of suspended cutoff walls is quite high and ranges between 0.13 and 0.30 [4]. Dam foundations composed of unstable sandy gravelly strata are especially prone to suffusion-induced erosion. This type of erosion is difficult to detect before the symptoms manifest on the outside of the dam foundations, by which time the level of erosion is often very severe. Although not publicly reported, severe piping erosion has been observed in several deep alluvium dam foundations in China, and this is an extremely difficult problem to manage. Suffusion erosion increases the permeability and size of the pore channels in the soil, thus changing the seepage fields of the dam foundations and increasing the seepage. Hence, suffusion erosion represents a hidden danger that threatens the safety of large dams. Alterations to the seepage fields of dam foundations, and erosion-induced increases in the compressibility of the soil will affect deformations in the cutoff wall and its overlying structure. Experimental studies on effects of this type have been reported in the literature [5,6,7,8,9]. The microscopic mechanisms of suffusion erosion and soil motion processes have also been simulated using discrete particle flow methods; however, suffusion processes on the scale of dam foundations are difficult to simulate using these methods owing to the limitations in the computational scale [10].

It is critical that a number of practical engineering questions related to suffusion erosion be addressed as soon as possible, for example, whether suffusion erosion will continue to develop within the dam foundations, whether the development of erosion will ultimately cause seepage failure in dam foundations, and whether limited levels of suffusion erosion will affect the structure of large dams. These are key issues in the engineering and design of dams on sandy gravelly alluvial strata.

To quantitatively describe the effects of piping erosion on the dam foundations and dam body along with the corresponding strains and deformations, a quantitative model describing the effects of piping erosion on the stress-strain relationships of soil is required. Since the loss of fine particles is an objective descriptor of suffusion erosion in soils, an experimental study was performed on the effects of fine particle losses on the stress-strain relationships of internally unstable sandy gravels, from which a constitutive model that included the particle loss indicator was constructed for soils undergoing suffusion erosion. In this work, triaxial tests and confined compression tests were used to study the effects of particle losses on the stress-strain relationships, and quantitative descriptive methods were proposed. Together, these provide a basis for quantitative assessments of stress and strain in dam bodies, and foundations that experience suffusion erosion.

## 2. The Effects of Fine Particle Losses on the Mechanical Properties of Soil

In soils where suffusion erosion has occurred, the coarse particles of the soil form a skeletal frame. This allows for fine particles to traverse the pore channels formed by these coarse particles under the influence of seepage. Generally speaking, the loss of fine particles will not necessarily lead to the collapse of the skeletal frame. During suffusion erosion, if the erosion of fine particles by seepage is not coupled with soil deformation, the volumetric content of the coarse particles and their arrangement/contact relationships will remain unchanged, although the soil porosity and the pores and spaces between the skeletal particles will increase as the erosion increases. For soils composed of large homogeneous particles, if the particles are maximally packed and small quantities of extremely fine particles are present in the spaces between the large particles, and then fine particle losses will only affect the seepage characteristics of the soil while having a minimal impact on the deformation characteristics. This particular circumstance may be observed in artificial rockfills. In natural sandy gravels, fine particles fill the spaces between the coarse particles. Thus, reductions in the number of fine particles will reduce the number of force transfer pathways that are mediated by fine particles, and will thereby increase the compressibility of the soil. Since coarse particles form the skeletal frame of a soil, it may be hypothesized that piping suffusion will not significantly alter the stress-strain characteristics, but may have an effect on the deformation moduli. An experimental study is therefore necessary to quantify these effects. As the erosion of soil in dam foundations varies in space and time, studies of the effects of the erosion level on the strength and deformation moduli of soils will be useful when simulating the effects of piping erosion on the deformation of dam foundations and dam bodies.

### 2.1. Experimental Plan

Triaxial and confined compression tests were performed on eroded and un-eroded sandy gravels to investigate the effects of particle losses on the strength and deformation characteristics/parameters of the soil. As it is difficult to obtain sandy gravelly samples that have been eroded by piping erosion with quantified levels of erosion, the samples used in this experiment were prepared with particle gradations and porosities equivalent to soils that have been eroded by piping erosion.

Based on the particle gradation and dry density of the natural samples, the characteristics of un-eroded samples were determined after similitude methods were used to remove the oversized particles. A list of ingredients for the un-eroded sample was thus obtained. To create samples that have been eroded by piping erosion, fine particles were removed from the list of ingredients used to prepare un-eroded samples, up to some weight corresponding to the desired quantity of fine particle loss. By creating samples in this manner, the volumetric content of the coarse particles was held constant, while the fine particle content was decreased in the eroded sample. This is generally consistent with the particle composition and porosity of soils that have been eroded by piping erosion. Triaxial tests and confined compression tests were then performed on samples with different levels of erosion, which revealed how the amount of erosion affected the stress-strain relationships of the soil.

In triaxial tests, when studying soil compressibility, it would be useful to record the deformation moduli during consolidation. However, due to the effects of membrane penetration, accurate measurements of volumetric deformation during isotropic consolidation are very difficult to perform. Since confined compression tests directly reflect the compressibility of the tested soil, they may be used to study the effects of fine particle losses on the volumetric deformation moduli of sandy gravels.

In this work, well-graded sandy gravels were used as the experimental material. The largest particles were 40 mm in diameter, and particles smaller than 5 mm accounted for 25.48% of the total weight of the natural soil. The specific gravity of particles larger than 5 mm was 2.83, while particles smaller than 5 mm had a specific gravity of 2.71. The eroded soil samples were prepared by removing fine particles (5 mm in diameter or smaller in accordance with the Standard [11]) from the list of ingredients for un-eroded soil samples, according to the desired ratio of erosion. The samples that had the same particle gradation and porosity as actual eroded soils with the same erosion ratio were thus prepared. Three sets of triaxial tests were performed, with the first set being performed on un-eroded soils. The dry density of the un-eroded samples was 2.36 g/cm^3^, while their porosity was 13.9%. In the second set of tests, the particles lost to erosion constituted 3% of the total mass, and the dry density and porosity of the resulting sample were 2.29 g/cm^3^ and 16.5%, respectively. In the third set of tests, the particles lost to erosion accounted for 6.5% of the total mass, and the dry density of the resulting sample was 2.21 g/cm^3^, while its porosity had increased to 19.5%. The triaxial test samples had a diameter of 150 mm and height of 300 mm, and saturated consolidated drained tests were performed. The samples for the confined compression tests had a height of 60 mm and a diameter of 100 mm. The confined compression tests were performed on un-eroded samples, and samples where fine particle erosion accounted for 3%, 6.5%, 10%, and 15%, respectively, of the total soil weight.

The moist tamping method was employed in this study. To avoid the fines migration during preparation of the sample, great care had to be taken to minimize disturbance. The automated experimental system used could conduct measurements and controls by computer through 16-bit A/D and D/A converters (AD1380KD, GEMAC, London, UK). The vertical load could be automatically applied by a motor-gear system at any rate. The cell pressure is applied by air pressure through an automatic air compressor (GA 5-11, Air Technologies, Columbus, OH, USA) regulated by E/P (Electronic to Pneumatic) transducers (550-AGD, Combine Co., Zhuhai, China). All of the pressure lines are connected to a draining system to remove any condensed water. The deviator load is measured by a submersible load cell (2022/2022-F, SENSY, Brussels, Belgium) mounted inside the cell. Axial displacement is measured externally by a Linear Variable Differential Transformer (LVDT) (GT2-P12, Keyence Corporation, Shanghai, China). Three pairs of clip gauges with the maximum capacity of ±0.002 m are employed to measure the radial strain.

The ratio between the amount of soil erosion and the total soil weight is henceforth defined as the erosion ratio. The erosion ratio differs from the volumetric erosion ratio, as the latter refers to the volume of the particles lost to erosion per unit volume. For soils with the same specific gravity in their soil particles, the volumetric erosion ratio = erosion ratio × (1 − initial porosity).

### 2.2. Results of the Triaxial Tests

**Figure 1:**
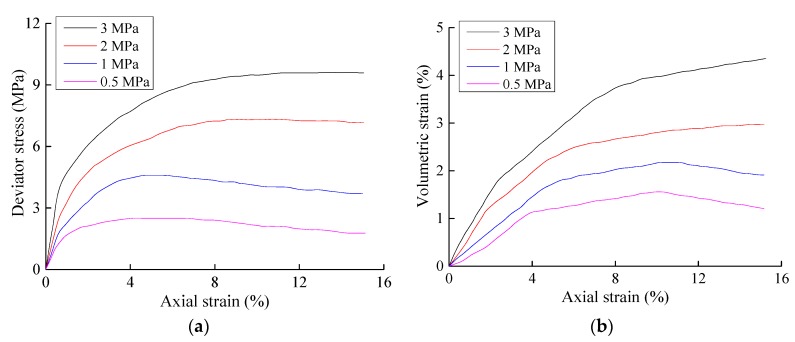
Results of the triaxial shear tests on the un-eroded samples. (**a**) Axial strain–deviator stress; (**b**) Relationship between the axial strain and volumetric strain.

**Figure 2:**
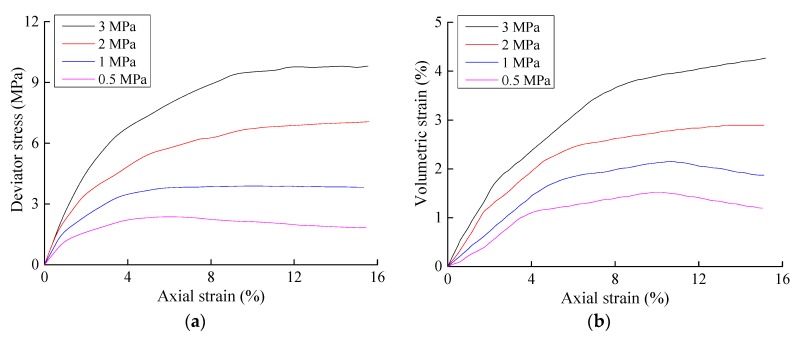
Results of the triaxial shear tests on samples with a particle erosion ratio of 3%. (**a**) Axial strain–deviator stress; (**b**) Relationship between the axial strain and volumetric strain.

**Figure 3:**
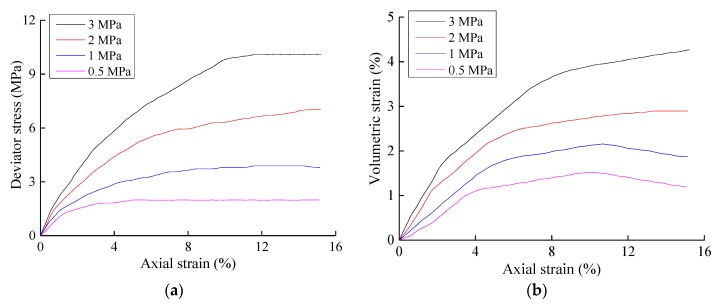
Results of the triaxial shear tests on samples with a particle erosion ratio of 6.5%. (**a**) Axial strain–deviator stress; (**b**) Relationship between the axial strain and volumetric strain.

The stress-strain relationship curves of the three conventional triaxial tests performed in this study are shown in Figure 1, Figure 2 and Figure 3, where *σ*_1_ represents the axial stress, *σ*_3_ represents the radial stress, *ε*_1_ represents the axial strain, and *ε_v_* represents the volumetric strain. The un-eroded soil sample and the soil samples with particle erosion ratios of 3% and 6.5% generally exhibited the same qualitative stress-strain characteristics. The strength of a soil decreases after erosion, and the magnitude of the soil strength decrease after particle erosion was quite significant. The shear modulus and bulk modulus also decreased significantly in that the magnitudes of the decreases in shear modulus were positively correlated with the confining pressure, whereas the magnitudes of the decreases in the bulk modulus were negatively correlated with the confining pressure. The decrease in shear modulus was also larger than that of the bulk modulus.

### 2.3. Results of the Confined Compression Tests

**Figure 4:**
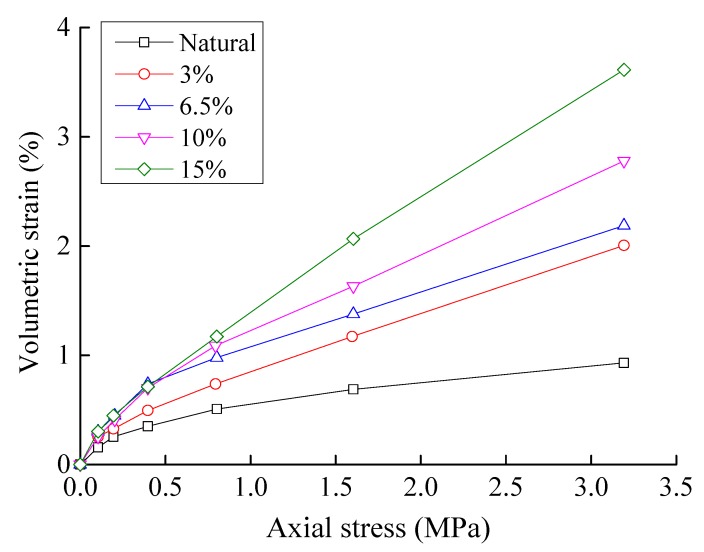
Compressive stress vs volume strain in the confined compression tests.

The sample preparation methods used for the confined compression tests were the same as those used for the triaxial tests, and all samples contained the same number of particles greater than 5 mm in size per unit volume. Samples with fine particle loss were obtained by reducing the content of particles smaller than 5 mm. The relationship between the axial pressure and volumetric strain is shown in Figure 4. In this figure, the natural sample and the samples with 3%, 6.5%, 10%, and 15% particle loss had porosities of 13.9%, 16.5%, 19.5%, 22.6%, and 26.9%, respectively. The results of this test demonstrate that the pores and gaps within the coarse particle skeletal frame increase as the particle losses increase, and the quantity of confined compression will also increase for some given pressure.

From the results above, the strength and deformation modulus of soil decrease as the particle losses increase. However, the stress-strain characteristics of the soil remain largely unchanged. Hence, the effects of the particle losses on the stress-strain relationships of soil can be described based on currently available models for describing the stress-strain relationships of sandy gravels in combination with currently available model parameters that are functions of the erosion ratio.

## 3. Effects of Particle Loss on Stress-Strain Relationships

The Duncan-Chang E-B model [12] is a simple model that can be used to provide a reasonable estimation of the stress-strain relationships of sandy gravels and rockfills. It is currently the most widely applied model in China for finite element analyses of rockfill dams because the parameters can be easily determined. In this section, this model will be used to study the effects of particle losses on the stress-strain relationships.

### 3.1. The Duncan-Chang E-B model

The definition of the tangent modulus of elasticity *E_t_* in the Duncan-Chang model is:

(1) $$E_{t}={\left[1-\frac{R_{f}(\sigma_{1}-\sigma_{3})}{{\left(\sigma_{1}-\sigma_{3}\right)}_{f}}\right]}^{2}E_{i}$$

where *σ*_1_ and *σ*_3_ are the principal stresses, (*σ*_1_ − *σ*_3_)*_f_* is the difference between the principal stresses when soil failure occurs, *R_f_* is a parameter, and *E_i_* is the initial tangent modulus.

According to the Mohr-Coulomb failure criterion:

(2) $${\left(\sigma_{1}-\sigma_{3}\right)}_{f}=\frac{2c\cos\varphi +2\sigma_{3}\sin\varphi }{1-\sin\varphi }$$

where *c* and φ correspond to the cohesion and internal friction angles of the soil, respectively.

The relationship between *E_i_* and *σ*_3_ is:

(3) $$E_{i}=kP_{a}{\left(\sigma_{3}/P_{a}\right)}^{n}$$

where *k* is the elastic modulus number, *n* is the elastic modulus exponent, and *P_a_* is the atmospheric pressure.

The equation in the Duncan-Chang model for the tangent modulus of elasticity contains five parameters: *k*, *n*, *R_f_*, *c*, and φ.

The tangent bulk modulus *B_t_* can be expressed as:

(4) $$B_{t}=dp/d\varepsilon_{v}$$

The equation for calculating *B_t_* in the Duncan-Chang E-B model is:

(5) $$B_{t}=k_{b}P_{a}{\left(\frac{\sigma_{3}}{P_{a}}\right)}^{m}$$

where *k_b_* is the bulk modulus number and *m* is the bulk modulus exponent.

The equation for calculating Poisson’s ratio in the Duncan-Chang E-B model is:

(6) $$\mu_{t}=\frac{1}{2}\left(1-\frac{E_{t}}{3B_{t}}\right)$$

Non-linear strength indices are often used for sandy gravels during the calculation of stress in high rockfill dams. In this case, *c* = 0, and the expression for φ is:

(7) $$\varphi =\varphi_{0}-\Delta \varphi \mathrm{lg}[\max(1,\sigma_{3}/P_{a})]$$

where $\varphi_{0}$ is the initial friction angle, and Δφ is the decrease in the friction angle when high pressures are present.

### 3.2. The Relationship Between the Erosion Ratio and Soil Strength

Table 1 shows the non-linear strength parameters of the 3% and 6.5% eroded and un-eroded soil samples that were obtained from the triaxial tests. Since only three sets of triaxial tests were performed in this study, the derivation of a more accurate relationship between the strength parameters and particle losses will be considered in a follow-up study.

### 3.3. The Effects of Particle Loss on the Deformation Modulus

Three sets of triaxial tests and confined compression tests were conducted to determine the relationship between erosion ratio and volumetric deformation. Table 2 lists the Duncan-Chang model parameters obtained from the three sets of triaxial tests. In this table, *k*, *n*, and *R_f_* are the shear deformation parameters, while *k_b_* and *m* are the volumetric deformation parameters.

When we obtained data from triaxial and confined compression tests, we analyzed the relationship between and the effects of the erosion ratio and volumetric deformation parameters.

#### 3.3.1. Relationship between the Erosion Ratio and Volumetric Deformation Parameters

Table 3 displays the calculations of the Duncan-Chang E-B model for the bulk modulus *B*. The regression parameters for the un-eroded sample were *k_b_* = 904 and *m* = 0.09, with a correlation coefficient of 0.5917, whereas the returned parameters were *k_b_* = 600 and *m* = 0.181, with a correlation coefficient of 0.8901 for the sample with 3% erosion, and *k_b_* = 282 and *m* = 0.32, with a correlation coefficient of 0.9722 for the sample with 6.5% erosion. Based on the relationship between the volumetric deformation modulus and confining pressure shown in Table 3, it can be seen that the Duncan-Chang model does not include the compression hardness of soils. It is very difficult to find patterns related to the effects of the erosion ratio on the volumetric deformation parameters from the three sets of *k_b_* and *m* volumetric deformation parameters shown in Table 2 as they do not reflect the patterns of volumetric deformation that occur following fine particle loss from sandy gravels. This is because the parameter regression based on the relationship between the volumetric strain and bulk stress in soils subjected to shear forces did not account for consolidation strain and stress, and thus is not representative. Therefore, it is very important to use the results of the confined compression tests to determine the relationship between volumetric compression and bulk strain.

Here, the Duncan-Chang E-B model will be analyzed from the perspective of the confined compression tests, to reveal the effects of erosion ratio on the parameters of the model. The compression pressure in the confined compression tests correspond to the minor principal stress *σ*_3_, which can be calculated as follows:

(8) $$\sigma_{3}=\;k_{0}F=(1-\sin\varphi )F$$

where *F* is the compression stress, *k*_0_ is the coefficient of lateral pressure, and φ is the internal friction angle of the soil material.

In this work, φ was assumed to be 40° in the lateral pressure calculations, and the lateral pressure coefficient was assumed to be 0.36. This may be used to convert the relationship of the compression stress and volumetric deformation into the volumetric stress-volumetric deformation relationship.

Table 4 describes the relationship between the segmented average bulk modulus and the maximum compressive stress in each segment, and it can be seen that the average bulk modulus of each segment decreases as the erosion ratio increases, i.e., the magnitude of the decreases was small when the pressure was low and large when the pressure was high. The median value of the loaded segment was assumed to be the stress level that corresponded to the segmented bulk modulus. A rough regression on the data in Table 4 then yields the volumetric deformation parameters for the Duncan-Chang E-B model for sandy gravels with different levels of erosion, as shown in Table 5. The results in this table demonstrate that *k_b_* and *m* decrease as the erosion ratio increases in a very consistent manner. These results validate the use of confined compression tests to study the relationship between the compressive deformation modulus and erosion ratio of sandy gravels.

**Figure 5:**
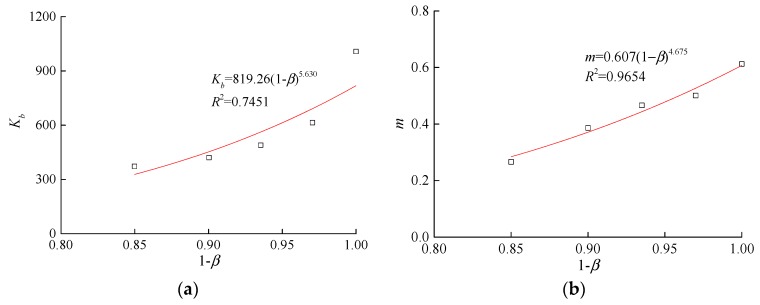
Relationship between the volumetric deformation parameters and erosion ratio. (**a**) *k_b_*-*β*; (**b**) *m*-*β*.

The fitting of the volumetric deformation parameters for the Duncan-Chang E-B model with the erosion ratios shown in Table 5 is shown in Figure 5. The fitting equation is as follows:

(9) $$k_{b}=a_{1}{\left(1-\beta \right)}^{b_{1}}$$

(10) $$m=a_{2}{\left(1-\beta \right)}^{b_{2}}$$

where *a*_1_, *b*_1_, *a*_2_, and *b*_2_ are the fitting parameters, which were 819.26, 5.630, 0.607, and 4.675, respectively, *β* is the erosion ratio, which is the ratio between the weight of the particles lost to erosion and the total weight of the initial particles.

#### 3.3.2. Relationship between the Erosion Ratio and the Shear-Deformation Parameters

Table 6 lists the hyperbolic regression parameters corresponding to the deviator stress–axial strain relationship of eroded and un-eroded soils. The correlation of the initial tangent modulus was very high, which indicates that the use of a hyperbola to simulate the axial strain-deviator stress relationships of conventional triaxial tests was appropriate. The *k* and *n* parameters in Table 2 were obtained using Equation (3) based on the initial tangent modulus, and it was found that the correlation coefficients of the three soil types were 0.864, 0.911, and 0.935. It can be seen that the Duncan-Chang model and its shear-deformation parameters provide a reasonable description of the axial strain–deviator stress behavior of a material. By comparing the two sets of tests, it was found that erosion significantly decreased the initial tangent modulus, and the amplitude of this decrease increased with the confining pressure. The *k* and *n* parameters also decreased substantially, and *R_f_* also exhibited some change.

As only three sets of triaxial tests were performed, the derived relationship between the shear modulus parameters and erosion ratios were not fully completed. Since the equations of the initial shear modulus and the volumetric deformation modulus are similar in form, the equations that relate the parameters of the initial shear modulus with changes in the ratio of erosion have the following forms:

(11) $$k=a_{3}{\left(1-\beta \right)}^{b_{3}}$$

(12) $$n=a_{4}{\left(1-\beta \right)}^{b_{4}}$$

**Figure 6:**
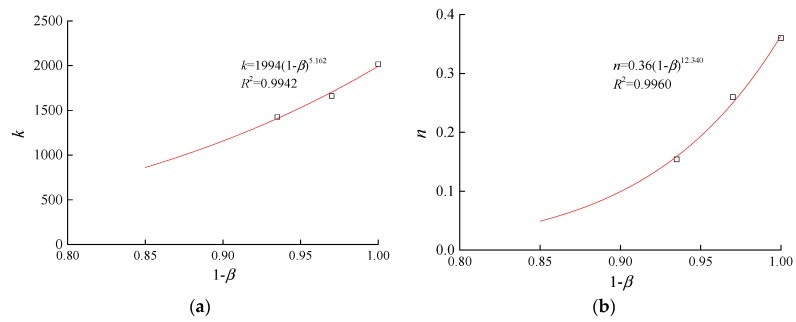
Relationship between the shear-deformation parameters and the erosion ratios. (**a**) *k*-*β*; (**b**) *n*-*β*.

In these equations, the fitting parameters *a*_3_, *b*_3_, *a*_4_, and *b*_4_ were 1994, 5.162, 0.36, and 12.34, respectively. Figure 6 indicates that the *k* and *n* parameters of the soil samples remained within the ordinary range of the parameters of the Duncan-Chang model for sandy gravels when the erosion ratio varied between 0 and 0.15.

There are ten piping erosion parameters that affect the deformation parameters of the Duncan-Chang model are *a*_1_, *b*_1_, *a*_2_*, b*_2_, *a*_3_, *b*_3_, *a*_4_, *b*_4_, *a*_5_, and *b*_5_. Each *a*_n_, *b*_n_ pair of parameters forms a set of influencing parameters for *k_b_*, *m*, *k*, *n*, and *R*_f_, respectively, and the deformation parameters for piping erosion in sandy gravel that were obtained in this experimental study are shown in Table 7.

The *R_f_* parameter primarily reflects the effects of the stress level on the shear modulus, and should decrease as the erosion ratio increases. Parameter *R_f_* is assumed to satisfy the following equation:

(13) $$R_{f}=a_{5}{\left(1-\beta \right)}^{b_{5}}$$

**Figure 7:**
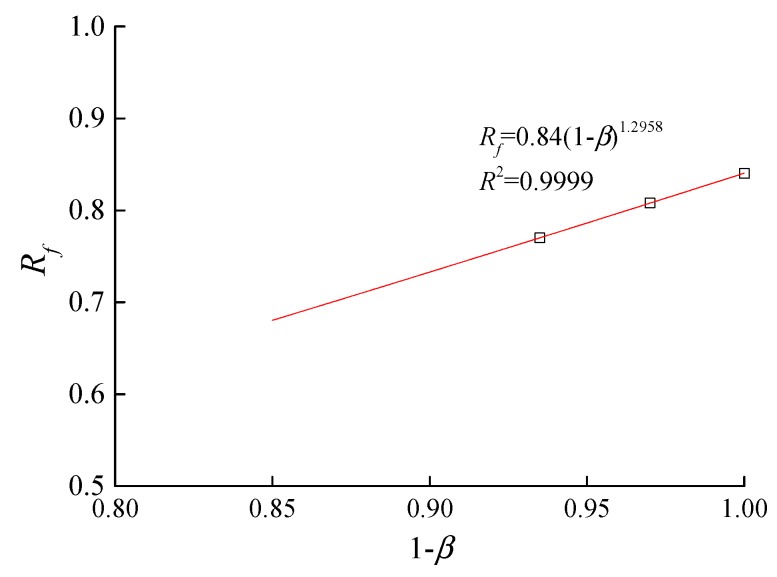
Relationship between the stress level parameter and erosion ratio.

In this equation, *a*_5_ and *b*_5_ are 0.84 and 1.296, respectively, and the results of the fit are shown in Figure 7.

If the deformation tests performed on un-eroded soils were more complete, the fitting equation should be made to pass through the erosion of 0 point during parameter fitting so that it converges to the un-eroded state during calculations on the effects of erosion.

## 4. Stress-Strain Relationships during the Processes of Suffusion

The calculation of soil strains during suffusion diffusion may be divided into two parts: (1) the increase in strain from the current stress state due to decreases (decay) in the deformation modulus of the sandy gravel; and, (2) the increase in strain caused by changes in the effective stress fields of the dam foundation, which were in turn caused by changes in the seepage coefficient of the eroded soil, or changes in external/internal loading.

(14) $$d\varepsilon_{ij}=d\varepsilon_{ij}^{0}+d\varepsilon_{ij}^{\sigma }$$

where *dε_ij_* is the total increase in strain, $d\varepsilon_{ij}^{0}$ is the increase in strain caused by deformation modulus decay, and $d\varepsilon_{ij}^{\sigma }$ is the strain induced by increases in stress. The additional strain induced by decreases in the deformation modulus may be calculated using the initial strain increment method. This is equivalent to the difference between the current and initial total strains of the soil after a time step is applied to the initial stress state. When the increment method is used in the calculations, the soil stress should be divided into *N* equivalent components and integrated to calculate the strain, since the deformation modulus is a tangent modulus. If we suppose that the stress of a unit element of eroded soil at the start of the time step is $\sigma_{ij}^{t0}$, then the increase in strain induced by changes in the deformation modulus under the initial stress state $d\varepsilon_{ij}^{0}$ may be calculated using the equation below:

(15) $$d\varepsilon_{ij}^{0}=\sum_{M=1}^{N}\left[D_{ijkl}^{-1}\left(\frac{(M-0.5)\sigma^{t_{0}}}{N},\beta_{t}\right)-D_{ijkl}^{-1}\left(\frac{(M-0.5)\sigma^{t_{0}}}{N},\beta_{t_{0}}\right)\right]\frac{\sigma_{kl}^{t_{0}}}{N}$$

where ***D****_ijkl_* is the tangent elastic matrix, *t*_0_ and *t* represent the start and end of the time step, and *i*, *j*, *k*, and *l* represent the axial variables of the coordinate with the subscript repetitions being indicative of summation operations, and *N* is the step number in the strain increment calculation.

The strain induced by the increase in stress $d\varepsilon_{ij}^{\sigma }$ may be calculated as follows:

(16) $$d\varepsilon_{ij}^{\sigma }=D_{ijkl}^{-1}(\frac{\sigma^{t_{0}}+\sigma^{t}}{2},\beta_{t})d\sigma_{kl}$$

Therefore, Equations (1), (3), (5), (6) and (9)–(16) form the stress-strain relationships of the Duncan-Chang E-B model for sandy gravels that are affected by piping erosion.This model and method of calculation are already being used in computational analyses on the stress and deformation caused by suffusion in dam foundations that use suspended cutoff walls, and in the design of several rockfill dams that are being built on alluvial strata of various thicknesses and depths. These will be described in further detail in a future article.

## 5. Conclusions and Outlook

In this work, the removal of fine particulate soils from structurally unstable sandy gravels was used to simulate soils with different levels of seepage-induced particle loss. Triaxial tests and confined compression tests were performed on samples with different levels of particle loss. The results demonstrate that particle losses only alter the stress-strain relationships of structurally unstable sandy gravels in a quantitative manner, without altering their qualitative characteristics. By constructing relationships between the model parameters and particle loss, we can calculate the deformation moduli of sandy gravels with erosion levels that vary over time and space. The stress-strain relationships of soils undergoing seepage-induced erosion can be described by dividing the increase in strain during this process into components induced by stress and decays in the deformation moduli. The Duncan-Chang E-B model was also used to construct quantitative methods, and equations and parameters for describing the effects of particle loss on stress-strain relationships were derived.

This is a preliminary attempt to quantitatively describe the effects of suffusion on the stress-strain relationships of sandy gravels, and the work performed in this research is still in its exploratory stages. We hope that our findings will inspire further work in this field of study. To satisfy the requirements of engineering applications, more experimental work is needed, and the models introduced in this work also require further development.

## Figures and Tables

**Table 1:** Strength parameters for the 3% and 6.5% eroded and un-eroded samples.

Sample	*ϕ*_0_ (°)	Δ*ϕ* (°)
Natural soil	53	9.9
Soil with 3% particle loss	50	6.1
Soil with 6.5% particle loss	46	4.8

**Table 2:** Duncan-Chang E-B model parameters of the soil samples.

Sample Type	*k*	*n*	*R_f_*	*k_b_*	*m*
Un-eroded soil	2017	0.36	0.84	904	0.090
3% eroded soil	1661	0.26	0.81	600	0.181
6.5% eroded soil	1427	0.154	0.77	282	0.321

**Table 3:** The calculation of the bulk modulus, *B*, from the results of the triaxial tests.

Confining Pressure (MPa)	0.5	1.0	2.0	3.0
Un-eroded soil	977	1174	1370	1071
3% eroded soil	655	845	1011	901
6.5% eroded soil	461	600	801	786

**Table 4:** Relationship between the segmented average bulk modulus and the compressive stress in the compressive tests.

Erosion Ratio (%)	*σ*_3_ (MPa)					
	0.36	0.72	1.44	2.88	5.76	11.52
0.0	333	688	1290	1332	2663	3590
3.0	229	688	688	917	1086	1101
6.5	235	356	356	938	1147	1131
10.0	184	369	491	590	851	786
15.0	195	382	389	523	516	594

**Table 5:** Volumetric deformation parameters obtained from the compressive tests.

Erosion Ratio (%)	*k_b_*	*m*
0	1003	0.61
3	613	0.501
6.5	487	0.466
10	423	0.386
15	372	0.266

**Table 6:** Hyperbolic regression parameters of the axial strain–deviator stress of soils.

Whether Erosion Occurred	Confining Pressure (MPa)	*Ei*	*R_f_*	Correlation Coefficient
Un-eroded sample	0.5	4099	0.86	1.000
	1.0	3912	0.75	0.996
	2.0	5283	0.84	0.999
	3.0	8021	0.90	0.999
Sample with 3% erosion ratio	0.5	3455	0.83	0.997
	1.0	3126	0.80	0.999
	2.0	4011	0.81	0.996
	3.0	6233	0.79	0.998
Sample with 6.5% erosion ratio	0.5	1902	0.81	0.998
	1.0	1906	0.82	0.999
	2.0	2272	0.79	0.999
	3.0	2465	0.65	0.996

**Table 7:** Influencing parameters of erosion in sandy gravels that affect the parameters of the Duncan-Chang E-B model.

*a*_1_	*a*_2_	*a*_3_	*a*_4_	*a*_5_	*b*_1_	*b*_2_	*b*_3_	*b*_4_	*b*_5_
819.3	0.607	1994	0.36	0.84	5.630	4.675	5.162	12.34	1.296
